# Cellular level lipidomics of two-dimensional cultures of adherent gut epithelial cell lines confirms a metabolic switch

**DOI:** 10.1039/d5an01183c

**Published:** 2026-04-27

**Authors:** Qianying Xu, Jake A. Penny, Emily Fraser, Federica Orsenigo, Matteo Barberis, Lee A. Gethings, Melanie J. Bailey, E. N. Clare Mills

**Affiliations:** a Division of Immunology, Immunity to Infection and Respiratory Medicine, School of Biological Sciences, Manchester Institute of Biotechnology, University of Manchester Manchester UK clare.mills@surrey.ac.uk; b School of Chemistry and Chemical Engineering, Faculty of Engineering and Physical Sciences, University of Surrey GU2 7XH Guildford UK; c School of Biosciences, University of Surrey Guildford UK; d Centre for Mathematical and Computational Biology, CMCB, University of Surrey Guildford UK; e Waters Corporation Wilmslow UK; f Department of Infectious Diseases, Guy's Hospital, King's College London London UK

## Abstract

*In vitro* cell models of the gut epithelium, particularly those based on the Caco-2 and HT29-MTX cell lines, play an important role in studying the uptake and metabolism of nutrients and pharmaceuticals. Previous studies using mass spectrometry imaging have shown a distinctive lipidome signature for these cells, alone and in coculture, although only limited information on lipid identities was obtained. A novel method employing limited proteolysis for sampling live, adherent cells using an automated capillary extraction workflow was developed which achieved single-cell sampling of Caco-2 cells although only clusters of HT29-MTX cells could be sampled due to mucus secreted by these cells. The lipidomes of the cell samples were mapped using LC-MS/MS and approximately 150 lipids were putatively identified. Further analysis of these data confirmed the distinctiveness of the Caco-2 and HT29-MTX cell lipidomes. Cell-to-cell heterogeneity was observed, especially in the Caco-2 cells, which may be indicative of variation in their differentiation state. Metabolic pathway analysis showed the distinctive lipidome of Caco-2 cells related to increased glycerol-3-phosphate pathway activity involved in di- and tri- glyceride synthesis. In contrast, HT29-MTX cells exhibited a more active phosphatidylcholine metabolism, related to their mucus-secreting capability. Future studies will explore wider application of the sampling procedure outlined here for single cell lipidomics of other adherent cell lines.

## Abstract 2

Advances in lipidomics analysis at a cellular level highlight important metabolic differences between Caco-2 and HT29-MTX cell lines which are widely used to study gut uptake and metabolism of nutrients and pharmaceuticals.

## Graphical abstract

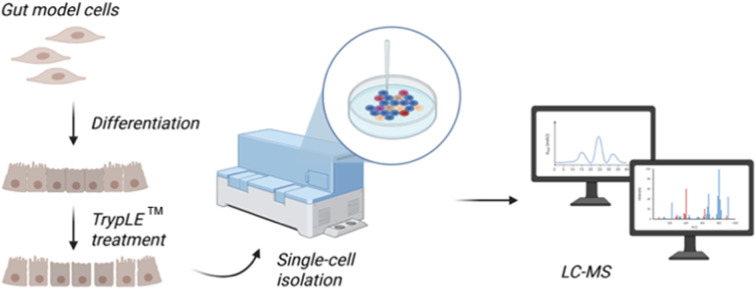

## Introduction

The colon cancer cell lines Caco-2 and HT29-MTX are commonly used as an *in vitro* 2-dimensional (2D) model of the intestinal absorptive epithelium (either cultured alone or in co-cultures), to assess the uptake of nutrients and pharmaceuticals.^1^ Despite their extensive use, gaps remain in our understanding of their metabolic status. This includes analysis of bulk cultures,^2^ although these may not represent how the cells behave in the 2D cocultures which mimic more closely the gut epithelium. An alternative methodology which can retain spatial information is mass spectrometry imaging (MSI) with single-cell resolution, which allows rapid lipidomic analysis of cell and tissue samples. One of the approaches uses matrix-assisted laser desorption ionisation (MALDI),^3,4^ whilst another employs desorption electrospray ionisation (DESI).^5^ We have recently applied DESI analysis to map the lipidomes of 2D cultures of Caco-2 and HT29-MTX cells (singly and in co-culture).^6^ This showed the cell cultures to be highly heterogeneous, with metabolic signatures of each cell type being clearly distinguishable.

However, MSI does not lend itself readily to quantification of individual lipid species, data which are required to more fully understand the different metabolic pathways that underly the metabolic signatures of these cells. Sampling approaches are now emerging which allow an in-depth profiling of such heterogeneity at a cellular level,^7,8^ which include conventional direct infusion or LC-MS/MS methodologies.^9,10^ However, it is challenging to build appropriate sample preparation workflows for single cell analysis, including isolation of single cell samples. To achieve this, a range of approaches have been applied based on different mechanisms including physical isolation methods, such as capillary picking^11,12^ and microfluidics^13,14^ which separate cells based on their shape or size. There are also biological separation techniques, including fluorescent-activated cell sorting (FACS) and magnetic-activated cell sorting (MACS), which use cell flow cytometry to isolate cells based on specific biological labels.^15,16^

Approaches, which allow single cell analysis, have the potential to further characterise the metabolic heterogeneity of Caco-2 and HT29-MTX cell line gut models identified using MSI. However, to date, such single cell level ‘omics information on gut cell lines is limited to Caco-2 cells.^17^ In this study, an automated capillary-based single cell isolation approach has been adapted to collect live cells Caco-2 and HT29-MTX cell lines from fully differentiated, adherent cultures. This approach has the advantage of sampling directly from two-dimensional cell cultures, to further characterise the heterogeneity of these cell models using liquid chromatography MS (LC-MS). Using this novel cell sampling method, we show that Caco-2 and HT29-MTX cell lines may be distinguished through a clearly defined, different lipid switch. Specifically, Caco-2 cells have higher levels of phosphatidyl cholines (PC) and lower phosphatidyl serines (PS) whereas HT29-MTX cells have higher levels of PS and lower levels of PC. Furthermore Caco-2 cells have higher levels of triglycerides (TGs) and lower diglycerides (DGs), the converse being so for HT29-MTX cell lines. These differences are broadly consistent with CaCo-2 cells acting as models of the enterocyte and HT29-MTX cells modelling goblet cells within the context of these both being immortal cell lines derived from colon cancer tissue.

## Materials and methods

All chemical reagents were either Optima or mass spectrometry (MS) grade unless otherwise specified. Caco-2 and HT29-MTX cell lines were purchased from ATCC® (LGC Group, Teddington, UK) and were used at passage no. 11 and 13 respectively, after thawing. Dulbecco's Modification of Eagle's Medium (DMEM) [high glucose], Dulbecco's Phosphate-Buffered Saline (DPBS), Foetal Bovine Serum (FBS) trypan blue solution (0.4% v/v), and EquiSPLASH lipidomix™ were purchased from Sigma-Aldrich (Dorset, UK). Sodium pyruvate, amphotericin B, penicillin/streptomycin, l-glutamine, TrypLE™ Express enzyme (1×) and 4′,6-diamidino-2-phenylindole dihydrochloride (DAPI) were purchased from Thermo Fisher Scientific (Hertfordshire, UK). Disposable C-Chip haemocytometer was from Labtech International Ltd (Heathfield, UK). Glass bottom µ-Dishes (35 mm) were from Ibidi GmbH (Thistle Scientific, Warwickshire, UK). All cell culture plasticware was purchased from either CellStar or Greiner Bio-One (Stonehouse, UK). MS Qsert vials were purchased from Waters Corporation (Wilmslow, UK) and 10 µm glass capillary tips were from Yokogawa (Tokyo, Japan).

### Cell culture

Caco-2 and HT29-MTX cells were bulk cultured in flasks with complete culture medium comprising DMEM supplemented with 10% (v/v) FBS, 2% (v/v) l-glutamine, 1% (v/v) sodium pyruvate, 1% (v/v) penicillin–streptomycin and 0.01% (v/v) amphotericin B. Cultures were maintained at 37 °C with 5% CO_2_ and passaged every 48–72 h upon reaching 80–90% confluence. For subculturing, cells were rinsed with DPBS to remove residual medium and incubated with TrypLE™ (∼2 mL per 75 mL flask) for 10–15 min to allow the detachment of cells. An equal volume of complete growth medium was then added to dilute the TrypLE™, and the resulting cell suspension was then re-seeded into fresh culture flasks at a density of 10^6^ cells per cm^2^.

For experimental cell sample preparation, each cell line was seeded into µ-Dishes at a density of 10^5^ cell per cm^2^. Cells were cultured for 21 days until fully differentiated as previously described,^6^ with media changed every 48–72 h throughout differentiation.

### Automated capillary single cell isolation

The medium of cells cultured in µ-Dishes was replaced with serum-free DMEM 2–3 h prior to sampling. Initial sampling trials showed Caco-2 monolayer tight junctions were sufficiently strong to prevent individual cells being sampled by the capillary which simply lifted the cells in sheets (SI Video S1). Since limited trypsin digestion is routinely used in the passage of both Caco-2 and HT29-MTX cells to disrupt tight junctions and allow cells to be removed from plasticware surfaces, this procedure was explored to allow sampling of individual cells. Different incubation times and levels of trypsin were used to allow cell sampling to be achieved. The final protocol that proved effective was the addition of 50 µL of TrypLE™ to each µ-Dish for 120 s for the Caco-2 cells and 3 min for the HT-29MTX cells, after which it was diluted by the addition of 50 µL serum-free DMEM. The culture dishes were placed in the sampling chamber of a SS2000 Single Cellome™ System (Yokogawa Corporation, Tokyo, Japan) which had been set-up at 37 °C with 5% CO_2_. Automated sampling of cells was performed using 10 µm glass capillary tips. Tips containing live cell samples were immediately frozen by placing either on dry ice or in a −80 °C freezer. Cells were stored at −80 °C until used for lipid analysis.

### LC-MS/MS analysis

To ensure representative analysis, at least 8 samples were analysed from each cell line. Each frozen cell sample was thawed and eluted into Qsert vial by flushing the microcapillary with 5 µL of the starting mobile phase A (60 (v/v) acetonitrile: H_2_O with 10 mM ammonium formate and 0.1% (v/v) formic acid) containing 16 ng mL^−1^ of EquiSPLASH standards, immediately prior to MS analysis as previously described.^18,19^ Samples were then injected onto Ultimate 3000 UHPLC (Thermo Fisher Scientific, USA) attached to a Q-Exactive Plus Orbitrap mass spectrometer (Thermo Fisher Scientific, USA). The HPLC was performed using an Accucore C30 column (2.6 µm, 150 × 2.1 mm, Thermo Fisher Scientific, UK), using a flow rate of 0.4 mL min^−1^. Lipids were eluted with a LC gradient was set to start with 30% of mobile phase B (85 : 10 : 5 (v/v) isopropanol : H_2_O : acetonitrile with 10 mM ammonium formate and 0.1% (v/v) formic acid) which was increased to 43% of mobile phase B after 5 min. The proportion of mobile phase B was further increased to 50% at 5.1 min, and raised to 70% at 14 min; it was then increased to 99% from 21 to 24 min, before being decreased back to 30% at 24.1 min and held for 4 min. The data was acquired in full scan in positive ionisation mode, with a heated ESI (HESI) probe at 320 °C with a spray voltage at 3 kV. The full scan automatic gain control (AGC) target was of 1 × 10^6^ with the mass range of 150–1200 *m*/*z*, and the detection resolution was set at 140 000.

### LC-MS data analysis

The raw data file was initially processed using MS-Dial (ver. 5.3.240719) using a mass tolerance of 0.01 Da as previously described.^9^ Lipid identification was performed using LipidBlast database with a mass tolerance of 0.01 Da for MS1, with an 80% identification score cutoff with [M + H]^+^, [M + NH_4_]^+^, and [M + H − H_2_O]^+^ as allowable adducts. For samples containing cell clusters, the abundance was averaged according to the number of cells per cluster (Table S1) prior to subsequent statistical analysis.

The statistical analysis such as PCA and heatmap was performed through MetaboAnalyst 6.0.^20^ Heatmap sample clustering was performed using Pearson correlation distance and Ward's hierarchical clustering model. Lipid metabolic pathway analysis was carried out using BioPAN (https://www.lipidmaps.org/biopan).^21^*Z*-Scores were used to assess regulation of the reaction pathway on the basis of lipid class. Using a statistical significance threshold of <0.5, equivalent to *Z* > 1.645.

## Results and discussion

### Cell isolation

**Fig. 1:**
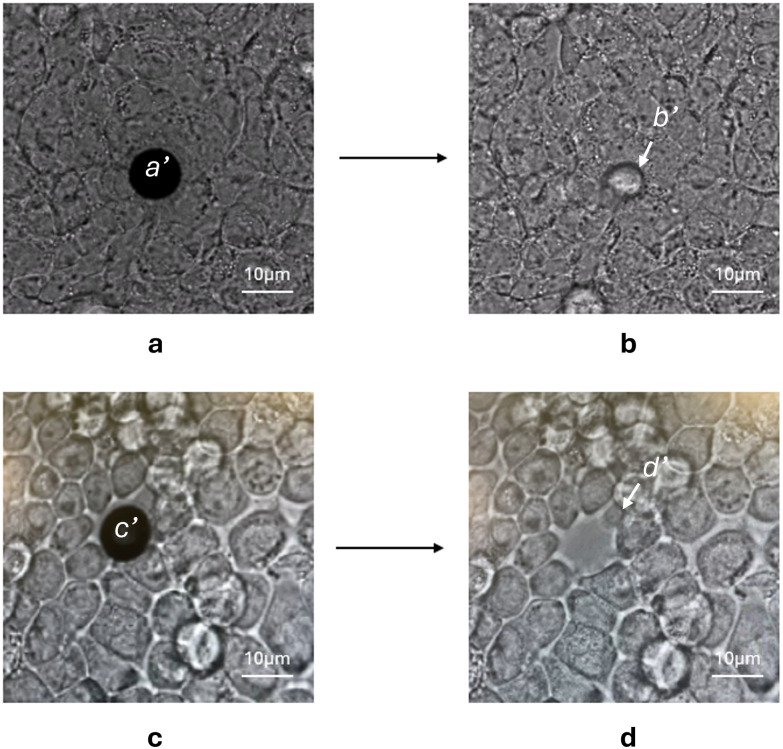
Caco-2 cell sampled by Yokogawa SS2000 platform. (a and b) bright field images of original untreated Caco-2 cell before (a) and after (b) capillary sampling. (c and d) images of a TrypLE™-treated Caco-2 cell before (c) and after (d) glass capillary extraction. The black disk labelled with a′ and c′ represents the position of sampling capillary; the b′ and d′ marks the location of individual cell sampled.

It was not possible to sample the adherent Caco-2 and HT29-MTX directly from fully differentiated confluent 2D cultures, due to the formation of tight junctions.^22^ As shown in Fig. 1a and b, when pressure was applied to the capillary with mechanical punction, instead of picking an individual cell it lifted the cell monolayer from the culture dish surface (see Figshare https://doi.org/10.48420/29840570.v2 to view).

**Fig. 2:**
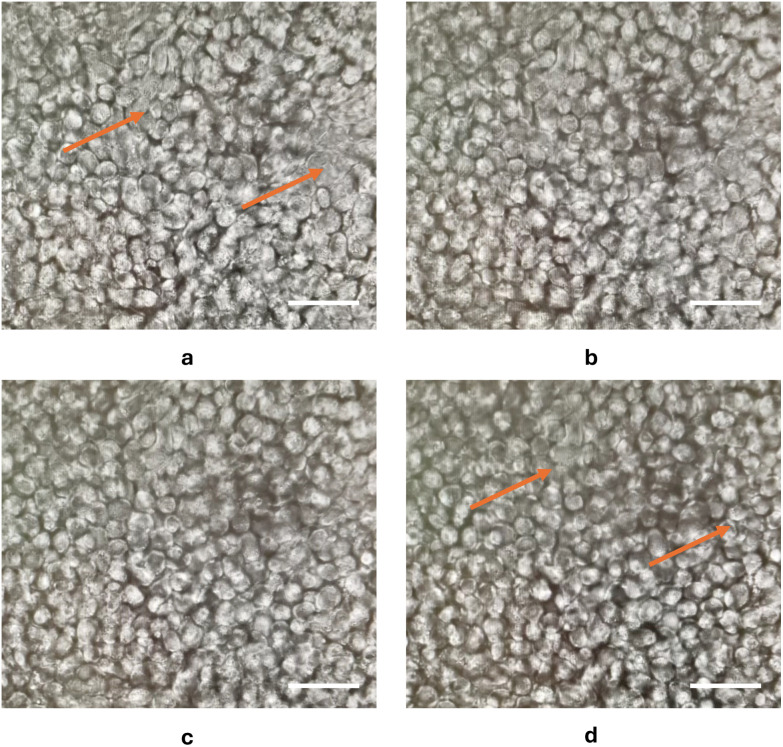
Morphological changes in Caco-2 cells exposed to low concentration of TrypLE, observed by light microscopy. Cells were incubated with TrypLE for (a) 0 s, (b) 40 s, (c) 80 s, (d) 120 s and visualised using a 20× objective. Scale bar = 50 µm.

To enable single cell sampling, a protocol was developed based on a modification of the techniques used to passage adherent cells using limited trypsin digestion.^23^ Only 20% of the TrypLE volume typically used in routine passaging was added, corresponding to ∼10 U mL^−1^ based on the supplier's specifications but using a much shorter exposure time than the 15 min used for cell passage. As shown in Fig. 2c and d, using very short time periods of exposure, the treatment had a negligible effect on the cell morphology. Thus, the Caco-2 cells took on a slightly more rounded appearance, and their boundaries became more defined after 120 s TrypLE incubation (Fig. 2a and d arrow-marked regions). Using this approach, it was possible to isolate individual live cells from the Caco-2 monolayer (Fig. 1c and d). This allowed individual cells to be sampled from within the spatial context of terminally differentiated Caco-2 cells, a state that they attain on reaching confluence. The protocol provided a time-limited window for cell sampling since after ∼1 h incubation in DMEM media the cells appeared to recover their tight junctions and were no longer tractable to capillary sampling (Fig. S1). This is a much shorter time period than the 20 h required for cell receptor recovery after cell passage following a 30 min incubation with another enzyme, accutase.^24^ These data suggests that the gentle treatment with TrypLE did not cause significant damage to the Caco-2 cells. Indeed, the level of trypsin employed is much lower than that found *in vivo* in the small intestine, where it has been estimated that the epithelium is exposed to 100 to 600 µg mL^−1^ of pancreatic trypsin,^25,26^ corresponding to a trypsin activity of 100 to 300 U mL^−1^.^27^ A slightly longer incubation time was required for the HT29-MTX cells which could only be successfully sampled after 3 min incubation with TrypLE.

Although single cell sampling of the Caco-2 cells was achieved in a reproducible manner this was more difficult to establish for the HT29-MTX cells which could be attributed to the mucus layer (SI Table S1). This affected both the ability of the microcapillary to sample cells through the mucus layer and posed challenges to visualisation of cells using light microscopy (SI Table S1). Such effects of mucus have been well described and indeed histological analysis of mucus producing epithelial cell surfaces is well described and many routine procedures remove the mucus layer in order to obtain clear micrographs.^28^ Consequently, the number of cells sampled was taken into account in the subsequent analysis.

### Caco-2 and HT29-MTX cell lipidomic analysis

**Fig. 3:**
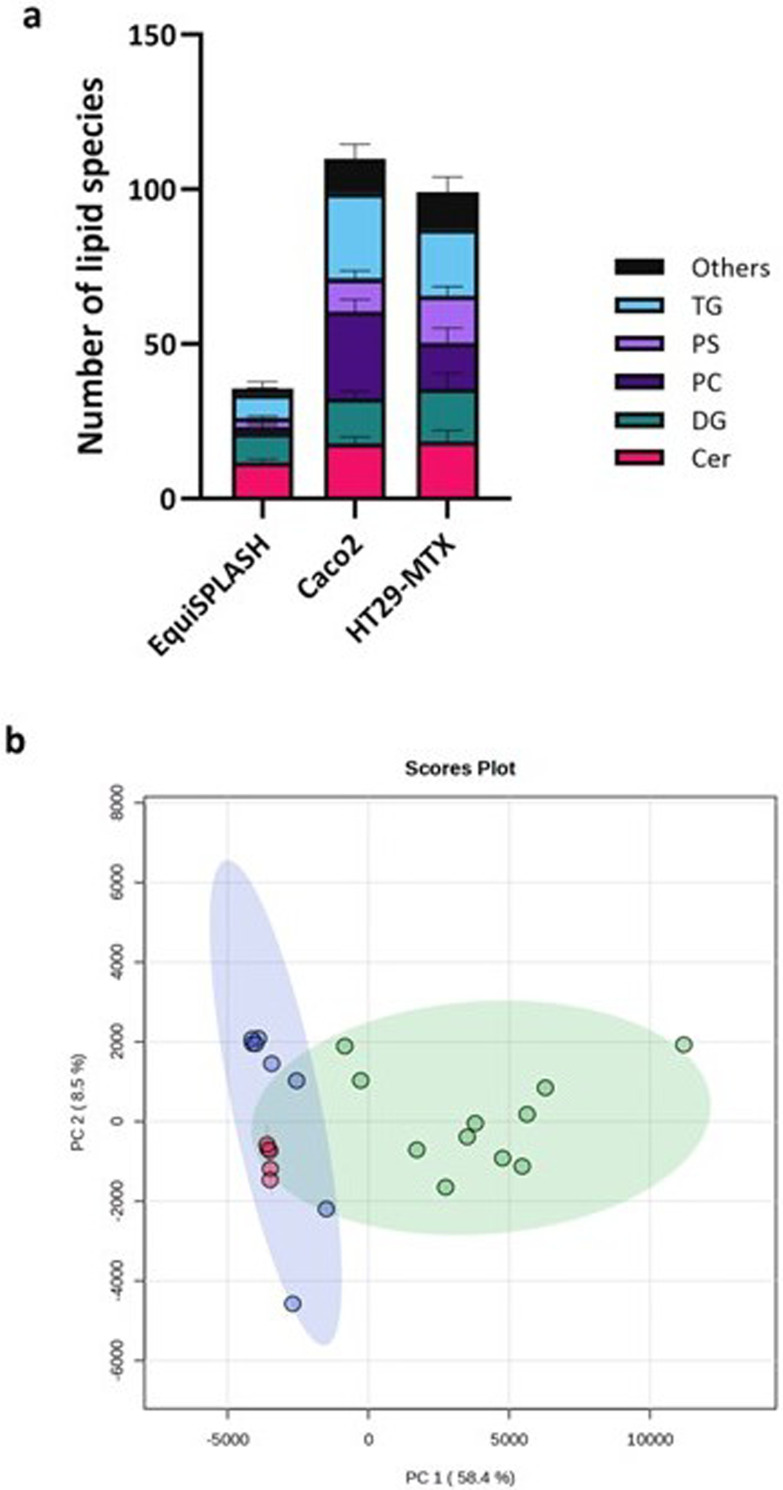
Lipidomic profiling of Caco-2 and HT29-MTX cells. (a) Lipid species coverage for EquiSPLASH, Caco-2 and HT29-MTX single cell samples. Lipid species including ceramides (Cer), diacylglycerols (DG), phosphatidylcholine (PC), phospholipid (PS), triglycerides (TG), and others that include sphingomyelins (SM), phosphatidylinositol (PI), phosphatidylglycerol (PG), phosphatidylethanolamine (PE), lyso-phosphatidylethanolamine (LPE), and lyso-phosphatidylcholine (LPC). (b) PCA plot of EquiSPLASH (*n* = 5, red) and cell samples of Caco-2 (*n* = 11, green) and HT29-MTX (*n* = 8, blue) cell lines. The confidence ellipses represent the 95% confidence interval for each group. The PCA plot with cell sample labels is shown in SI Fig. S2.

Around 150 lipids were assigned across all cell samples, indicating the sensitivity and feasibility of the single-cell level LC-MS based lipidomics (Fig. 3a). In comparison, approximately 30 lipids were detected in the EquiSPLASH lipid standard, which served both as internal reference and as a basis for class-based filtering of lipid species. The PCA plot (Fig. 3b and SI Fig. S2) showed the cell types could be distinguished based on their lipidome based on PC1 which accounted for 58.4% of the variance. The EquiSPLASH lipid standard replicates were clustered much more tightly, but were well-separated from cell sample lipids, providing a reference and that analysis of cell derived lipids is valid (Fig. 3b). Gut epithelial cell samples showed greater variance than the lipid standard, and whilst some of this may arise from technical issues, particularly cell sampling, subcultures of these isolated tumour cell lines are known to be variable.^29,30^ Indeed this impacts on the reliability and reproducibility of data obtained with these cell models, especially in toxicological studies.^31^ Less is known about the variability of HT29-MTX cells but given the fact that lipids (notably phosphatidyl cholines) are associated with the secreted mucus,^32,33^ it is likely that the mucus associated with the cell samples further contributes to the heterogeneity observed. The variation in Caco-2 and HT29-MTX *in vitro* cell models has also been attributed to different stages of differentiation^34,35^ which may also account for the variability in TGs, observed in the Caco-2 cells. Although the differences observed in the cell types in the current study were observed in DESI imaging experiments^6^ they differ from reported analysis of bulk cell cultures of Caco-2 cells and another sub-clone of the HT29 cells.^2^ Thus, Rombout and coworkers reported the lipidome of these cell types resembled each other more closely and were more similar to tumour tissue than was observed in the current study. However, it is not clear whether Rombout and co-workers cultured Caco-2 cells using a protocol where they are terminally differentiated, a process which is critical to their attaining a more enterocyte-like phenotype.

The predominant lipid class assigned in the single-cell samples were diglycerides (DGs), followed by triglycerides (TGs), with the latter being more prominent in the Caco-2 cells (Fig. 3a; SI Data Sheet 1). Over 50% of the lipids identified were also identified in previous DESI analyses^6^ (SI Data Sheet 2). Other important lipid species putatively identified were phosphatidyl cholines (PCs) and phosphatidyl serines (PSs).

**Fig. 4:**
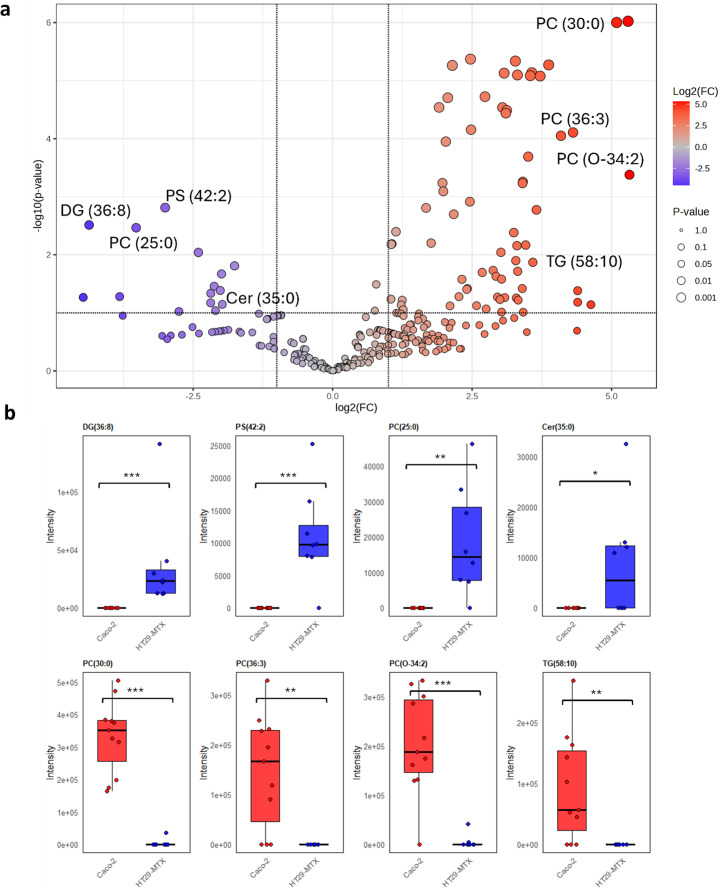
Comparison of the relative abundance of lipids in Caco-2 and HT29-MTX cells. (a) Volcano plot showing the fold changes in the lipid abundance between Caco-2 and HT29-MTX, each point represents a lipid feature. A more fully annotated plot is provided in SI Fig. S3. (b) Representative examples of four upregulated and four downregulated lipid features selected from the volcano plot. Each point corresponds a single-cell sample, with red indicating Caco-2 cells and blue indicating HT29-MTX cells. * 0.01 < *p* < 0.05, ** *p* < 0.01, *** *p* < 0.001.

Volcano plot analysis shows the differential lipid expression between Caco-2 and HT29-MTX (Fig. 4 and SI Fig. S3). The *x*-axis represents the log2fold change, where positive values indicate higher abundance of lipid species in Caco-2 cell samples and negative values indicates higher abundance in HT29-MTX cell. The *y*-axis is the −log (*p* value), indicating statistical significance (Fig. 4a). It was notable that the most abundant lipids in the HT29-MTX cells were diglycerides, such as DG (36:8) as were certain phospholipids, such as PS (42:0), PS (42:2) together with certain ceramides, such as Cer (35:0) (Fig. 4b). Other lipid species including PCs [*e.g.* PC(O-34-2), PC (30:0) and PC(36:3)] and TGs [*e.g.* TG(58:10)], were significantly upregulated in Caco-2 cells (Fig. 4b).

**Fig. 5:**
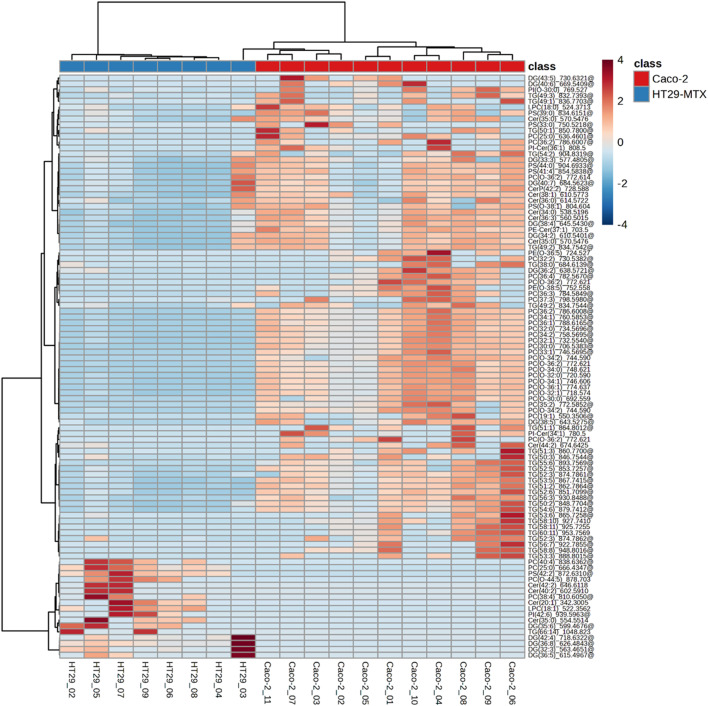
Hierarchical clustering analysis of the lipid profiles of Caco-2 and HT29-MTX cell samples. Clustering of the top 100 features identified in the Volcano plot (Fig. 4B) to be differentially abundant between Caco-2 (purple) and HT29-MTX (green) single cells. Columns represent individual cells, and rows correspond to distinct lipid species.

Heatmap analysis using unsupervised hierarchical clustering was undertaken to compare the lipidomes across the individual cell samples (Fig. 5 and SI Fig. S4). As expected, the cells grouped primarily according to their cell type, indicating the reproducibility and distinctive nature of the lipid profile of each cell line. Regarding the clustering lipid species, similar features between Caco-2 and HT29-MTX was observed compared with volcano plot. Lipid classes, such as PCs and TGs, were found in Caco-2 cells.

Variation in lipid composition was observed across the replicate samples of each cell type. This heterogeneity was evident in PCs, such as PC (O-34:2) and TGs in Caco-2 cells, DGs and ceramides in HT29-MTX cells (Fig. 4b). Commonalities and differences in the lipid composition of the two cell lines were highlighted which were consistent with those identified in the volcano plot. Thus, lipid classes, such as PCs and TGs, were enriched in the Caco-2 cells, which is consistent with the lipid packaging function of intestinal epithelial cells. In contrast, HT29-MTX cells showed increased PC synthesis, which is consistent with their ability to synthesis and secret mucus, mucin granules being enriched in phospholipids, more than 90% of which consist of PCs and LPC.^36,37^ This heterogeneity was also evident in PCs, such as PC (O-34:2) and TGs, in Caco-2 cells, and DGs and ceramides in HT29-MTX cells (Fig. 4b). Since the number of samples analysed between Caco-2 and HT29-MTX cell types was unequal, a randomised subset *t*-test analysis was performed to check the reliability of these observations. The lipids that remained significant across these subset groups with selection frequency over 60% and which remained significant after Welch's *t*-test and false discovery rate correction (Fig. S6) closely matched those found with the full dataset, including those highlighted in Fig. 4b, demonstrating these findings are robust.

Heatmaps were also generated to illustrate the variance between individual cells of the Caco-2 cell line using either the top 50 or 100 most variable features (SI Fig. S5). This was only done for the Caco-2 cells since sufficient single cells were sampled. This analysis showed the lipid types contributing most to inter-cell heterogeneity were predominantly TGs and PCs, including TG (60:11), TG (52:3) and PC (36:3). A possible biological explanation for this variability is that Caco-2 cells were captured at different stages of differentiation. Caco-2 cultures are known to progress from a stem-cell like states to enterocyte-like cells over several weeks.^34^ During this transition, acyl-CoA-dependent lipid metabolism is dramatically upregulated, which results in increased synthesis of TGs and PCs.^38^ Thus, it is plausible that at least part of the inter-cellular variability observed in these lipid classes may result from variation in the differentiation state of individual Caco-2 cells within the population as a whole.

**Fig. 6:**
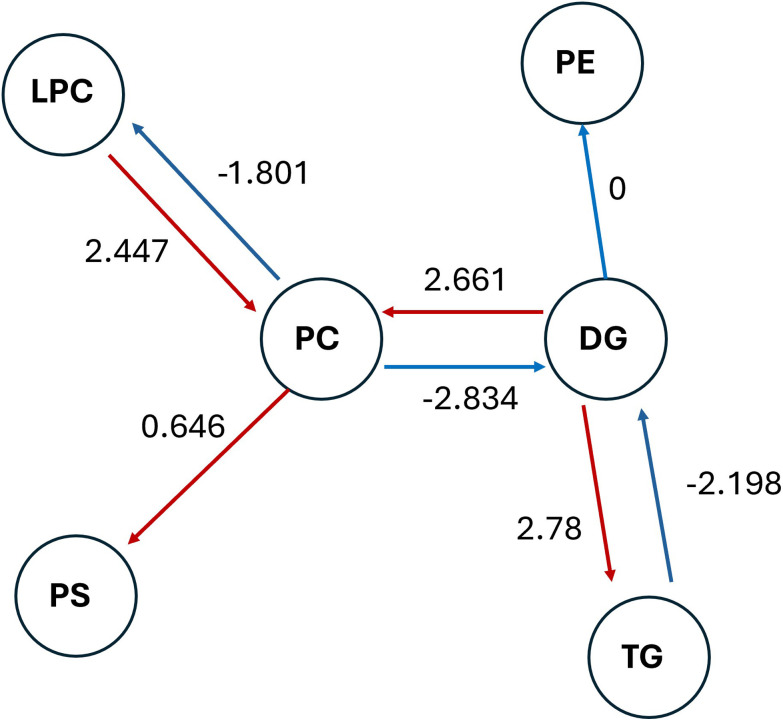
Comparative lipid pathway analysis of Caco2 and HT29-MTX.cell lines. Nodes represent lipid class (PC, DG, TG, LPC, PS, PE) and arrows represent predicted metabolic conversions. Digits on the arrows indicate log2fold changes in lipid abundances (Caco-2 *vs.* HT29-MTX). Positive values indicate upregulation in Caco-2, while negative values indicate relative enrichment in HT29-MTX, with colours to visualise the direction of conversion in the model, with colours help to visualise the direction of conversion in the model. Further details were listed in Table S1.

To better understand the lipid signatures that underlie the metabolic switch between the two cell types, a comparative network analysis of the lipid species was performed (Fig. 6). The analysis indicated that the conversions from LPC to PC (log_2_ FC: 2.447), DG to PC (log_2_ FC: 2.661), and DG to TG (log_2_ FC: 2.78) were strongly upregulated in Caco-2 cells, suggesting enhanced membrane synthesis and lipid storage activity.^36,37^ In contrast, conversions from PC to DG (−2.834) and TG to DG (−2.198) were downregulated, indicating reduced lipid breakdown in Caco-2 cells compared to HT29-MTX cells. These observed fold changes map onto the glycerol-3-phosphate pathway, particularly regarding the conversion of DGs into TGs and PCs, which is known to dominate the synthesis of TG in terminally differentiated Caco-2 cells.^38^ This is because this cell line lacks the enzyme monoacylglycerol acyltransferase, which is highly expressed in human intestine where the monoacylglycerol I-pathway is also important.^39^ The observations in this report are also consistent with expression analysis which has confirmed that Caco-2 cells possess higher levels of many proteins involved in the uptake of fatty acids and fat droplet formation, compared to HT29-MTX cells.^40^ Thus, Caco-2 cells, like enterocytes, are polarised and express fatty acid-binding proteins (FABPs), allowing them to take up free fatty acids from culture media.^36,37^ These fatty acids are first converted to diglycerides through the glycerol-3-phosphate pathway, and then esterified together with fatty acyl-CoA, to form TGs.^41^ These neutral lipids are normally stored in cellular lipid droplets, playing a crucial role in maintaining cellular lipid homeostasis. The higher level of TGs in Caco-2 cells is consistent with the lipid packaging function of intestinal epithelial cells involved in chylomicron formation, structures which allow lipid transport of lipids and some fat-soluble nutrients such as vitamin A, around the body. Chylomicrons are known to be secreted by differentiated Caco-2 cells and are rich in TGs and other lipids like phospholipids and cholesterol ester.^41^ In contrast, HT29-MTX cells showed increased PC synthesis, which is consistent with their ability to synthesise and secret mucus, mucin granules being enriched in phospholipids, more than 90% of which consist of PCs and LPC.^32,41^

## Conclusions

This study establishes a workflow using capillary sampling of cells from adherent confluent cultures which achieved single-cell level analysis for one of the cell lines studies, Caco-2. Although the TrypLE treatment resulted in only minimal morphological changes observed using light microscopy and rapid recovery was observed based cells regaining their adherent phenotype, further detailed characterisation of the impact of such treatment on cell-surface markers using techniques, such as immunofluorescence, are needed to further validate and optimise the approach.

Subsequent LC-MS/MS analysis allowed identification of ∼150 lipid species, showing that the sampled cells had a lipidome consistent with the known phenotypes of Caco-2 and HT29-MTX cells, with the major lipid classes comprising DGs, TGs, PCs and PS's. Differences between Caco-2 and HT29-MTX cells revealed distinct lipid metabolic signatures, with Caco-2 cells showing enriched TG synthesis and storage, while HT29-MTX cells exhibited elevated PC levels, reflecting their respective roles in lipid absorption and mucus secretion. Out data on single cell samples of Caco-2 cells presented in this work are consistent with previous DESI-MS analysis^6^ although differ from the analysis of bulk cell culture.^5^ Single cell sampling of the mucus-producing HT29-MTX cells proved more challenging with clusters of cells being sampled. Nevertheless, the lipid signature of these samples was both distinctly different from the Caco-2 cells and was also consistent with previous DESI analysis. The distinctive lipidomes of Caco-2 and HT29-MTX cells are also consistent with microscopic analyses showing the formation of nuclear lipid droplets and lipid associated promyelocytic leukaemia structures in Caco-2, but not HT29 cells.^42^ These observations indicate that the gentle tryptic digestion protocol used to allow cell sampling did not cause significant metabolic disruption. This approach reveals both intercellular diversity and cell line-specific lipid phenotypes at a single-cell resolution, and warrants further validation of this method, linking expression analysis and sampling from co-cultures, and suing other types of adherent cell lines. This will pave the way to investigating the metabolome of individual cell types in complex investigating the metabolome of individual cell types in complex tissue samples, thus monitoring molecular metabolism during differentiation or disease progression at the single-cell level.

## Author contributions

Q. X. and C. M. conceptualised and designed the work. F. O. contributed to cell culture procedures. Q. X. performed the experiments with the input from E. F. on the cell sampling workflow optimisation and undertook the data analysis with J. P. J. P. carried out the mass spectrometry data acquisition. Ma. B. interpreted the lipidomic data and formulated the metabolic switch underlying the work. L. G. and Me. B. provided technical resources and oversight of the analysis. Q. X. drafted the manuscripts with input from Ma. B. and C. M. All authors have reviewed, provided comments on the manuscript and approved the final manuscript.

## Conflicts of interest

J. P., E. F. and Me. B. are now employed at Kings College London. Q. X. is currently employed by the University of Surrey on a Knowledge Transfer Partnership project with Waters Corporation but undertook the current work as a PhD student at the University of Manchester. LG is an employee of Waters Corporation, a vendor of mass spectrometers. All other authors have no interests to declare in relation to this submitted work.

## Supplementary Material

AN-151-D5AN01183C-s001

AN-151-D5AN01183C-s002

## Data Availability

The mass spectrometry data sets have been deposited in the MetaboLights repository reference MTBLS14074; additional data are also available at Figshare.^43^ Supplementary information (SI) is available. See DOI: https://doi.org/10.1039/d5an01183c. This includes the following Figures and Tables: Table S1: Cell picking log; Table S2: Lipidomic pathway differences between Caco-2 and HT29-MTX cells; Figure S1: Micrograph of Caco-2 cells 1h TrypLE treatment; Figure S2: Annotated PCA plot; Figure S3: Volcano plot showing the fold changes in the lipid abundance between Caco-2 and HT29-MTX with detailed annotation of each lipid feature; Figure S4: Hierarchical clustering heatmap top 100 (a) and top 50 (b) features with highest variance between individual Caco-2 cell samples; Figure S5:  Differential lipid distribution between Caco-2 and HT29-MTX cells after subset test.
