# Supplementary material for: Cellular level lipidomics of two-dimensional cultures of adherent gut epithelial cell lines confirms a metabolic switch
Source: Analyst. 2026 Apr 27;151(11):3267–75. doi: 10.1039/d5an01183c (PMC13125980; doi:10.1039/d5an01183c)
Supplement: AN-151-D5AN01183C-s002 [file AN-151-D5AN01183C-s002.pdf]

Supplementary Information (SI) for Analyst.
This journal is © The Royal Society of Chemistry 2026

Cellular level lipidomics of two-dimensional cultures of adherent gut epithelial cell lines confirms a metabolic switch

Qianying Xu[a†], Jake Penny[b†], Emily Fraser[b], Federica Orsenigo[c], Matteo Barberis[a,d], Lee Gethings[a,c,e], Melanie Bailey[b,f] Clare Mills[a,c]

[a]*Division of Immunology, Immunity to Infection and Respiratory Medicine, School of Biological Sciences, Manchester Institute of Biotechnology, University of Manchester, Manchester, United Kingdom*

[b]*School of Chemistry and Chemical Engineering, Faculty of Engineering and Physical Sciences, University of Surrey, GU2 7XH, Guildford, United Kingdom.*

[c]*School of Biosciences, University of Surrey, Guildford, United Kingdom.*

[d]*Centre for Mathematical and Computational Biology, CMCB, University of Surrey, Guildford, United Kingdom.*

[e]*Waters Corporation, Wilmslow, United Kingdom*

[f]*Department of Infectious Diseases, Guy's Hospital, King's College London, London, UK*

†These authors contributed equally to this work.

Correspondence should be sent to Professor Clare Mills clare.mills@surrey.ac.uk

Contents

Table S1: Cell picking log 1

Table S2. Lipidomic pathway differences between Caco-2 and HT29-MTX cells 3

Figure S1. Micrograph of Caco-2 cells 1h TrypLE treatment. 4

Figure S2: Annotated PCA plot 5

Figure S3. Volcano plot showing the fold changes in the lipid abundance between Caco-2 and HT29-MTX with detailed annotation of each lipid feature. 6

Figure S4. Hierarchical clustering heatmap top 100 (a) and top 50 (b) features with highest variance between individual Caco-2 cell samples. 7

Figure S5. Differential lipid distribution between Caco-2 and HT29-MTX cells after subset test. 8

Table S1: Cell picking log

Cell Sample	Comment	No of cells
**Caco2 cells**		
CaCo_02_01	One cell plus one cell body. Clean pick. Stained with Hoechst 1:20,000 to allow validation of cell picking.	Two
CaCo_02_02	Two cells	Two
CaCo_02_03	One cell confirmed on Z stack	One
CaCo_02_04	One cell, clean pick, confirmed on Z stack	One
CaCo_02_05	Three cells, confirmed on Z stack.	Three
CaCo_02_06	One cell, clean pick.	One
CaCo_02_07	One cell., confirmed on Z stack.	One
CaCo_02_08	One cell.	One
CaCo_02_09	One cell	One
CaCo_02_10	One cell	One
CaCo_02_11	One cell, confirmation by video and initial Z stack.	One
**HT-29MTX cells**		
HT29_01	Not clear cell pick; data excluded from analysis	None
HT29_02	Multiple cells – difficult to confirm by microscopy due to mucus.	Four
HT29_03	Single cell although microscopic confirmation difficult to establish due to mucus	One
HT29_04	~ Eight cells.	Eight
HT29_05	~ Three cells plus debris.	Three

HT29_06	Difficult to estimate cell numbers due to mucus	Ten
HT29_07	~ Four cells	Four
HT29_08	~ Ten cells, including detached floating cells.	Ten
HT29_09	~ Eight cells	Eight

* The number of cells collected by the capillary was noted during at the time of collection and and subsequently verified against the recorded sampling video for the best estimation.

**Sample HT29_01 has been excluded from the analysis as no cell was sampled.

Table S2. Lipidomic pathway differences between Caco-2 and HT29-MTX cells

Reaction chains	Z-score
**Lipid subclass active reaction**	
DG→TG	3.135
LPC→PC	2.426
DG→PC→LPC	2.106
DG→PC→PS→PE	1.73
**Lipid subclass suppressed reaction**	
PC→LPC	1.92
PC→PS	1.901
TG→DG	1.836
**Lipid species most active reaction**	
DG(35:3) → TG(53:5)	2.937
DG(34:2) → PC(34:2) → PS(34:2)	2.589
LPC(18:0) → PC(36:2)	2.234
DG(34:4) → TG(52:6) → DG(34:6)	1.885
DG(36:2) → PC(36:2)	1.835
DG(38:7) → TG(58:8)	1.773
**Lipid species most suppressed reaction**	
PC(O-36:1) → PS(O-36:1)	3.104
LPC(18:0) → PC(38:1)	1.929

*Generated from BioPAN[1]

BioPAN is a pathway analysis tool powered by LipidMAPs. It compares the relative abundances of the molecules or lipid classes provided between two experimental conditions when mapping to the lipidmaps database. When the imported lipid species mapped to

known enzymatic transformation in the lipidmaps database, Biopan calculated the log-transformed abundances between product and substrate species across conditions, presented as Z-score, which considers the mean and standard deviation. This Z-score represents how strongly the observed substrate-product lipids deviates from the expected direction under no pathway changes:

- A **positive Z-score** indicates relative accumulation of products compared to substrates, suggesting pathway activation.
- A **negative Z-score** indicates relative accumulation of substrates compared to products, suggesting pathway suppression.
- Z-scores near zero indicate no consistent directional change.

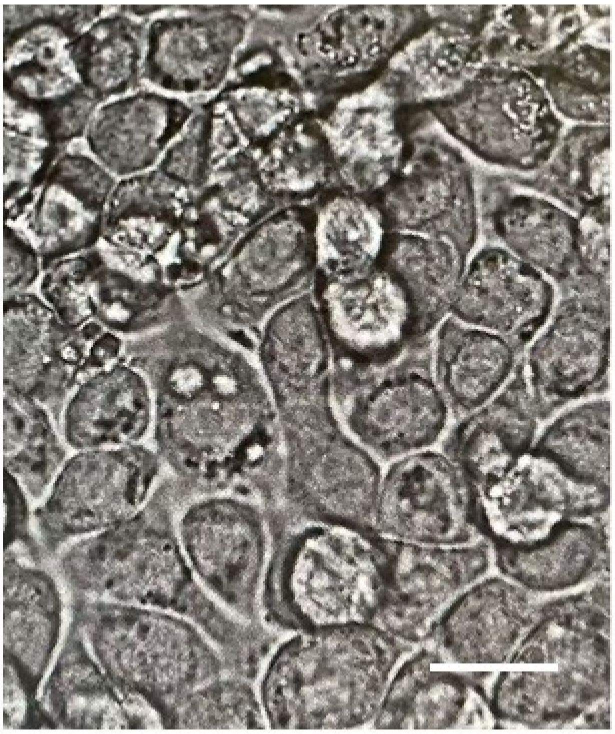

Figure S1. Micrograph of Caco-2 cells 1h TrypLE treatment.

Cells were incubated at 37°C in SS2000 sampling chamber supplemented with 5% CO_2. Scale bar = 50 µm.

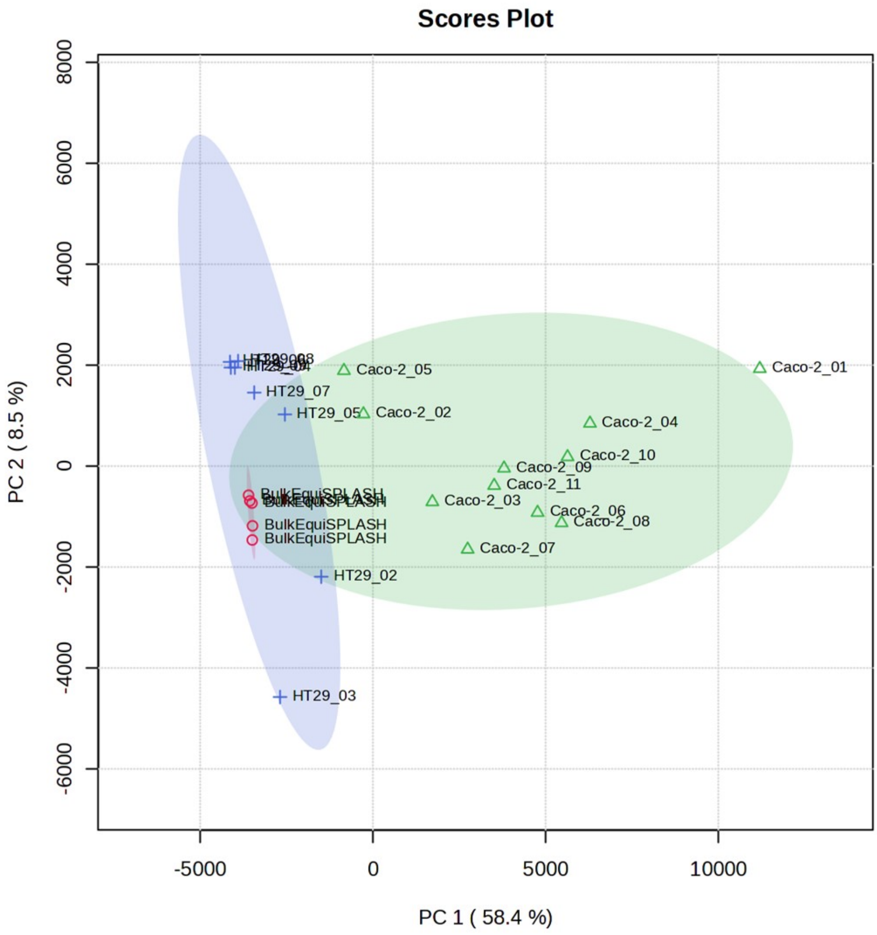

Figure S2: Annotated PCA plot

EquiSPLASH (n=5, red) and single-cell samples of Caco-2 (n=11, green) and HT29-MTX (n=8, blue). Cells are marked with sample number as described in Supplementary Table S1**.**

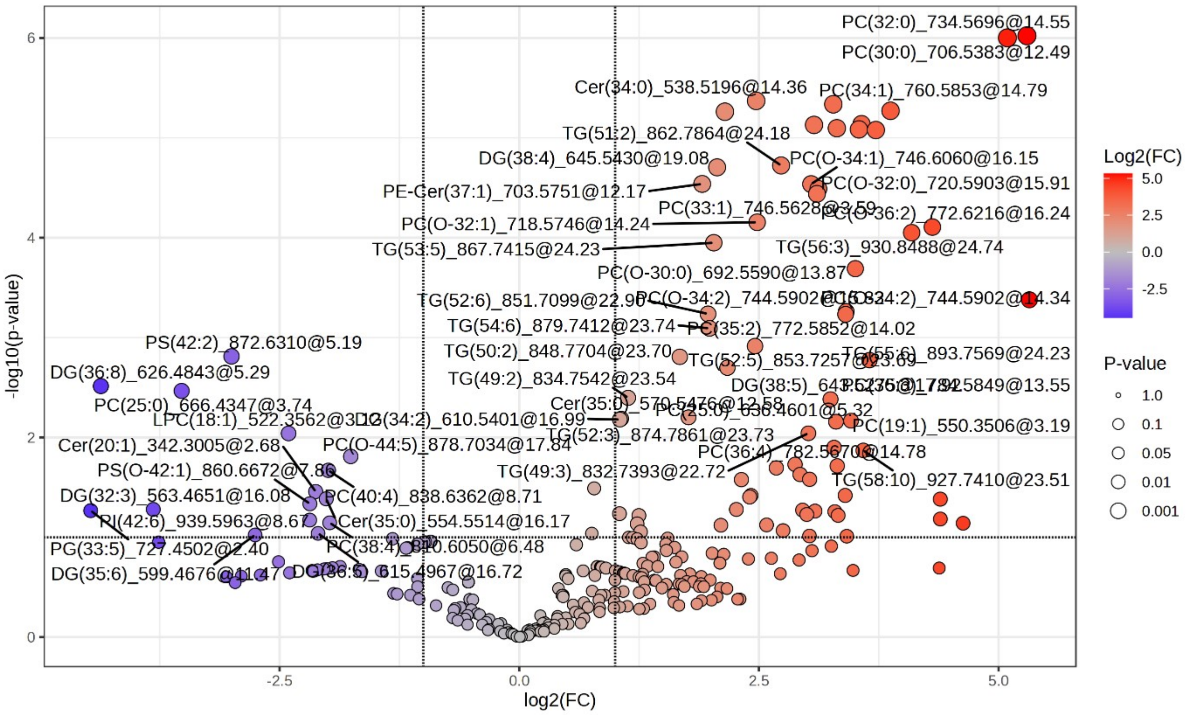

Figure S3. Volcano plot showing the fold changes in the lipid abundance between Caco-2 and HT29-MTX with detailed annotation of each lipid feature.

Lipid features are represented by coloured circles.

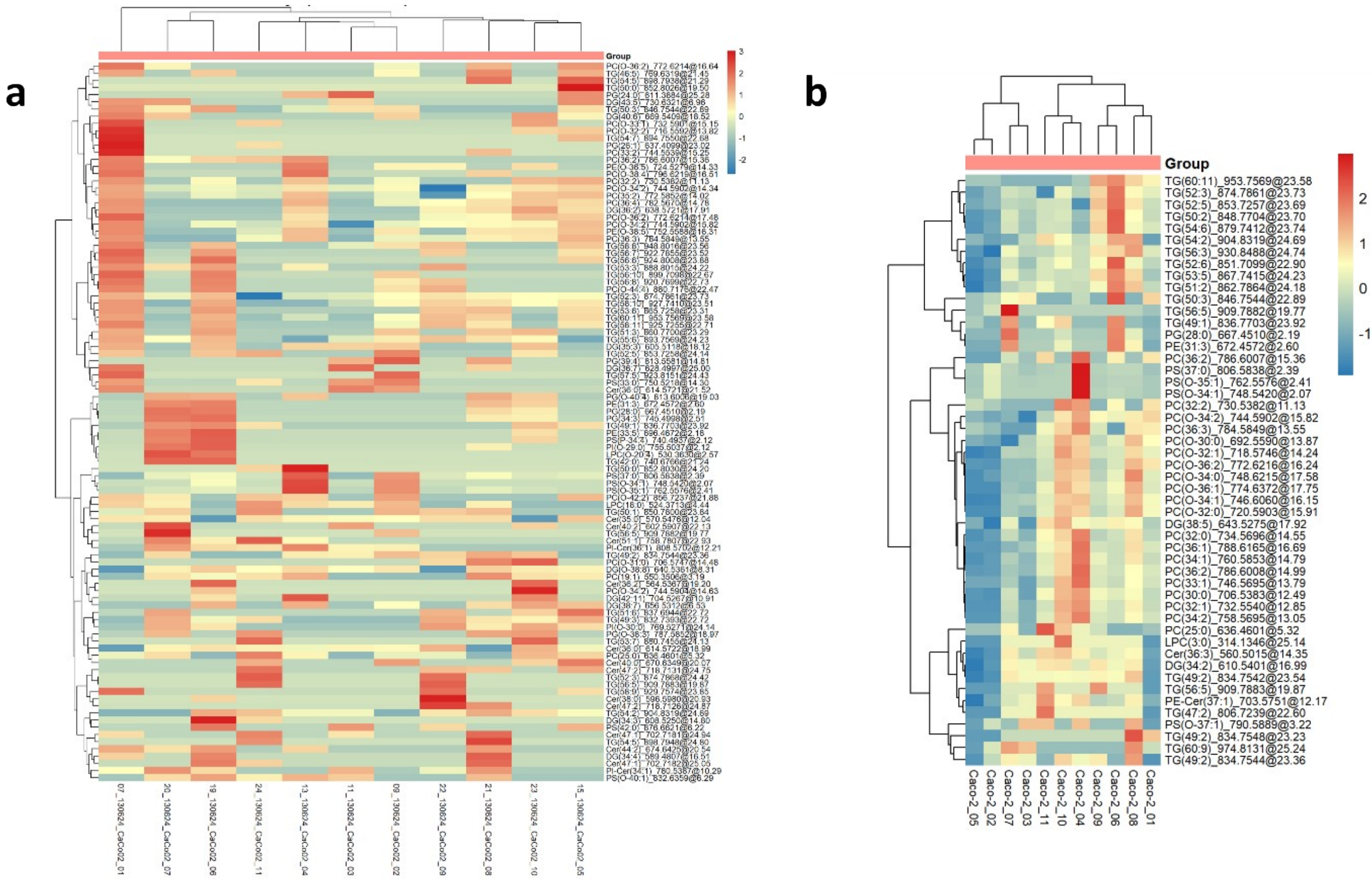

Figure S4. Hierarchical clustering heatmap top 100 (a) and top 50 (b) features with highest variance between individual Caco-2 cell samples.

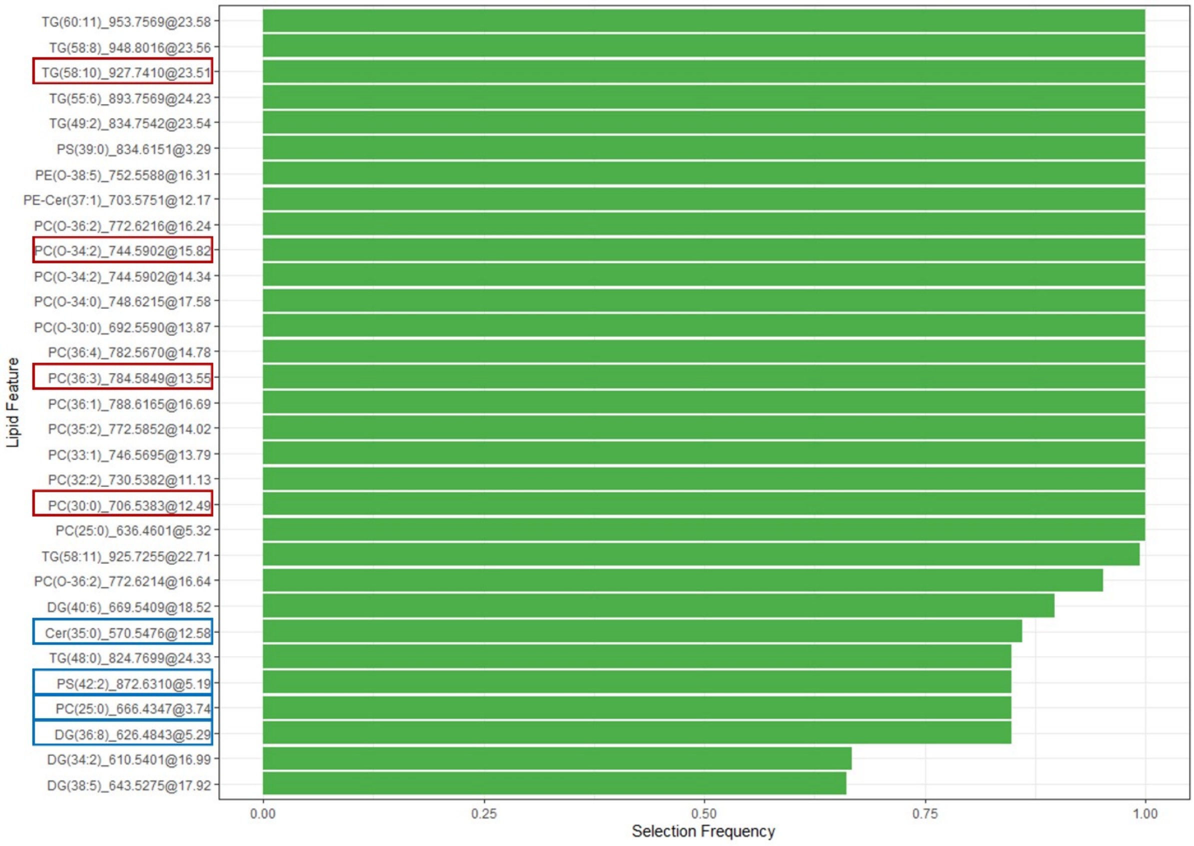

Figure S5. Differential lipid distribution between Caco-2 and HT29-MTX cells after subset test.

Selection frequency represents the proportion of all 165 combination of Caco-2 sub-samples in which the lipids remained significant after Welch's t-test and false discovery rate correction. Highlighted lipids in brackets indicating the corresponding lipids identified from the full dataset for Caco-2 (red) and HT29-MTX (blue) cells in Figure 4b.

References

1. C. Gaud, B. C. Sousa, A. Nguyen, M. Fedorova, Z. Ni, V. B. O'Donnell, M. J. Wakelam, S. Andrews and A. F. Lopez-Clavijo, F1000Research, 2021, 10, 4.
